# Ibuprofen Can Induce Syndrome of Inappropriate Diuresis in Healthy Young Patients

**DOI:** 10.1155/2013/167267

**Published:** 2013-06-12

**Authors:** Céline Roche, Céline Ragot, Jean-Luc Moalic, Fabrice Simon, Manuela Oliver

**Affiliations:** ^1^Department of Biochemistry, Laveran Military Teaching Hospital, 4 Boulevard Laveran, 13384 Marseille Cedex, France; ^2^Department of Biochemistry, Legouest Military Teaching Hospital, 57700 Metz Cedex, France; ^3^Department of Infectious Diseases and Tropical Medicine, Laveran Military Teaching Hospital, 4 Boulevard Laveran, 13384 Marseille Cedex, France

## Abstract

A 30-year-old caucasian woman, without past medical history or known drug use, was admitted to the emergency department for persistent fever and arthralgias. The laboratory analysis showed moderate hypoosmolar hyponatremia (Na: 132 mmol/L, osmolality: 239 mOsm/L), normal sodium excretion (<20 mmol/L), and a high urinary osmolality (415 mOsm/L). Later, she deteriorated with seizures and deeper hyponatremia (Na: 113 mmol/L) and so was moved to the critical care unit. At first, no obvious aetiology was found, the patient was euvolemic, as she was well hydrated and lacked concerning findings of heart failure, renal disease, or liver cirrhosis. A syndrome of inappropriate diuresis (SIAD) was proposed, and corrective measures were started immediately to reduce her hyponatremia, including restriction of fluid intake. The administration of intravenous hypertonic saline solution permitted normal neurological status to be restored and corrected the sodium concentration but induced reversible acute renal failure. Further investigation revealed that the patient had ingested 8 g ibuprofen two days before admission. After other aetiologies were ruled out, drug-induced SIAD due to ibuprofen was the most likely diagnosis for this patient. SIAD-associated hyponatremia and acute renal failure are rare side effects of nonsteroidal anti-inflammatory drugs, particularly in young people. Therefore, this case may represent a unique case of NSAID-induced SIAD and highlight the need to obtain thorough medication histories and exclude all other potential causes in hyponatremic patients.

## 1. Introduction

The syndrome of inappropriate diuresis (SIAD) is the most common disorder of sodium and water balance [1]. It is a diagnosis of exclusion and must be distinguished from several other types of hyponatremia because each requires different management. The most common causes are increased hypothalamic production of ADH, due to neuropsychiatric disorders, drugs, pulmonary diseases, HIV infection, or ectopic production of ADH which occurs in carcinoma, lymphosarcoma, or leukaemia.

Ibuprofen is a nonsteroidal anti-inflammatory (NSAID) drug used to treat moderate pain and/or fever. The recommended dose varies with body mass and indication. A dose of 400 mg to 1200 mg per day (i.e., 17 to 36 mg/kg) is considered the maximum amount for over-the-counter use [2]. Despite a very wide use, ibuprofen is rarely associated with severe toxicity. Drugs as NSAIDs cause SIAD by enhancing the action of ADH at the renal tubule level. However, the proportion of drug-induced SIAD resulting from NSAID is less than 0.5% [3]. An idiopathic form of the syndrome has been reported, but poorly documented. Also, 5% of SIAD is thought to be idiopathic [4] and is a discrete category of SIAD [5]. Maybe a proportion of these are actually ibuprofen induced. In the USA, from 1988 to 1990, 50 614 intoxications with NSAIDs were described [3], predominantly with ibuprofen. 131 patients (0.26%) had major outcomes with 10 deaths (drug overdose). In Great Britain in 2003, enquiries about the management of patients possibly suffering from ibuprofen overdose account for over 5% of the total enquiries received by the London Centre of the UK National Poisons Information Service. In each of the 1033 cases, there were complicating factors related to other drugs and/or other diseases [6]. Regarding the cases described in the literature, elderly and comorbid patients develop drug-induced SIAD more than young people. We report a rare case of a 30-year-old healthy and euvolemic woman who developed severe hyponatremia and acute renal failure after absorption of 152 mg/kg ibuprofen. After a thorough investigation and exclusion of more common causes, SAID was thought to be drug induced.

## 2. Case Presentation

A 30-year-old woman was admitted to the infectious diseases unit for fever and nausea (without vomiting) evolving over 5 days and resistant to analgesic and antipyretic drugs (acetaminophen). She was not anorexic, had no significant past medical history, under no medication, and she denied any medical or toxic ingestions. Temperature was 37°C, pulse rate was 100 b/min, and blood pressure was 150/70 mmHg. Physical examination did not reveal any abnormality, and EKG and chest X-ray were normal. Laboratory analysis only showed a mild biological inflammation (c-reactive protein: 8 mg/L) and a moderate hyponatremia (132 mmol/L). The results for the remaining biological tests were in the normal range (potassium: 3.8 mmol/L, chlorine: 96 mmol/L, urea: 2.8 mmol/L, and creatinine: 55 mmol/L). Urinalysis showed no abnormality and additional urine toxicological testing exclude drugs abuse. The next day, she complained of nausea, headache, and diffuse pain evocative of meningism.

The cerebrospinal fluid was acellular without biochemical abnormality. A brain MRI excluded an intracranial bleed or other focal abnormality, whereas an EEG showed signs of encephalopathy. Biochemistry tests were within normal limits, except serum sodium levels (113 mmol/L) and osmolality (239 mOsm/L). Natriuresis and urinary osmolality were preserved (137 mmol/L and 415 mOsm/L, resp.). On day 2, despite normal saline perfusion (2 litres in 24 hours) and presumptive antiinfective treatments (amoxicillin, ceftriaxone, and aciclovir), the patient convulsed and was transferred to intensive care unit. Her natremia fell to 105 mmol/L on day 3 (osmolality: 231 mOsml/L). Cortisol and adrenocorticotrophic hormone levels were normal (random cortisol and ACTH stimulation test); aldosterone levels were 3 times higher than normal and vasopressin (ADH) was within normal limits (2.39 pg/mL). Fluid restriction was undertaken and sodium levels reached 124 mmol/L in 24 hours. While neurological disorders were improving, acute renal failure occurred (creatinine levels rose from 36 to 259 *μ*mol/L, BUN rose to 6.9 mmol/L), so fluid restriction was stopped. On day 6, the patient was apyretic and renal function was restored with natremia reaching 132 mmol/L. Several aetiologies were ruled out (viral, bacterial, and toxic, including heavy metals), and there was no evidence of psychogenic polydypsia (nursing staff did not report any excessive drinking). She had no known cause of antidiuresis. There was no argument for hypervolemia (congestive heart failure, cirrhosis, or nephrosis), hypovolemia (haemorrhage, gastroenteritis, or diuretic abuse), renal failure, or adrenal failure. Thyroid function tests and chest radiography gave normal results. Neuropsychiatry disorders were excluded, as well as pulmonary diseases (tuberculosis or lung abscess), HIV infection, meningitis, encephalitis, and ectopic production of ADH (carcinoma, Hodgkin's disease …). Further investigations led to the discovery that the patient had ingested more than 20 tablets of ibuprofen 400 mg (representing 152 mg/kg) for back pain in the two days before admission to hospital. The outcome was favourable and the patient left hospital 12 days later.

## 3. Discussion

Hyponatremia is a common disorder, usually more prevalent in older adults [7]. SIAD is the most common form of normovolaemic or dilutional hyponatremia. The diagnosis of SIAD is based on five criteria [8]: hypotonic hyponatremia (natremia < 135 mmol/L), persistent urinary sodium excretion (>30 mmol/L), plasmatic osmolality < 280 mOsmol/L, urine osmolality in excess of plasma osmolality, absence of oedema, or volume depletion, and normal renal, adrenal, or thyroid function [1]. Clinical features are nonspecific, mainly neurological, and can threaten life. The severity of symptoms is related to both the absolute serum sodium concentration and its rapid decrease, particularly if greater than 0.5 mmol/L/h, which most likely explains the seizure activity in our patient. The management of SIAD patients may include efficient treatment of the primitive disease and removal of excess total body water. Fluid restriction remains the safe mainstay of hyponatremia management, as applied to our patient. Unfortunately, a convulsive episode associated with severe hyponatremia (113 mmol/L) occurred, requiring its rapid correction, as recommended by most guidelines [9, 10].

Inappropriate antidiuresis is usually due to the administration or endogeneous production of ADH, causing renal water reabsorption and resulting in hyponatremia with increasing extracellular fluid volume. Endogenous production can be either eutopic or ectopic, induced by a wide variety of diseases (cancers, neurological disorders, and lung diseases), drugs, or injuries (Table 1). Whereas some drugs are implied in ADH stimulation (carbamazepine and vincristine), other drugs (NSAIDs and chlorpropamide) enhance its action at the renal tubule level [11] or play a role in prostaglandin inhibition (such as ibuprofen). For the reported patient, we ruled out many causes of SIAD after analysis of her history, physical examination, and initial medical record. Ibuprofen-induced SIAD was the most likely diagnosis despite her young age. NSAIDs can adversely affect the kidney because they can induce sodium retention and antagonize the action of diuretics, impair free-water clearance, and cause hyponatremia [12]. Similar cases have been described in the past, but the patients suffered from chronic diseases such as advanced chronic renal failure [13], von Willebrand's disease [14], or were extremely old (>80 y) [15].

Whereas over-the-counter antipyretics and/or analgesics are relatively safe for adults, cyclooxygenase inhibitors may induce a wide variety of side effects [16], which usually concern elderly patients or are found in critical situations such as hypovolemia or anesthesia. Except for newborns [17], acute renal failure is rarely described in the literature [18, 19], but the low report of cases is probably underestimated since renal failure is often moderate and diuresis preserved. The Naranjo criteria classify the probability that an adverse event is related to drug therapy based on a list of weighted questions, which examine factors such as the temporal association of drug administration and event occurrence, alternative causes for the event, drug levels, dose-response relationships, and previous patient experience with the medication. In our case report, the score was five, so the adverse drug reaction was classified as probable. But the Naranjo criteria do not take into account drug-drug interactions. Moreover, other causes as back pain, an undiagnosed viral infection, are also plausible explanations of what happened.

This case stresses the importance in asking about history specifically pertaining to analgesics as commonly patients may not be open about the use of these medications because they are over-the-counter, easily accessible and viewed to be benign ingestions by the general public.

## 4. Conclusion

This clinical case stresses the importance that clinicians should give to recognising NSAIDs as a potential cause for patients who present with SIAD, even where there are no risk factors such as extreme age or renal failure.

## Figures and Tables

**Table 1:** Most common causes of SIADH.

*Increased hypothalamic production of ADH *	
Neuropsychiatric disorders:	
(i) infections: meningitis, encephalitis, abcess	
(ii) vascular: thrombosis, subarachnoid or subdural haemorrhage, temporal arteritis, stroke	
(iii) neoplasm: primary or metastatic	
(iv) psychosis, delirium tremens	
(v) other: Guillain-Barré syndrome, acute intermittent porphyria, autonomic neuropathy …	

Drugs:	
(i) intravenous cyclophosphamide	
(ii) carbamazepine	
(iii) vincristine or vinblastine	
(iv) haloperidol	
(v) bromocriptine	
(vi) general anaesthesia	
(vii) nicotine …	

Pulmonary disease:	
(i) pneumonia: viral, bacterial, fungal	
(ii) tuberculosis	
(iii) lung abscess, empyema	
(iv) acute respiratory failure	
(v) positive pressure ventilation	
(vi) other: asthma, pneumothorax …	

Postoperative patient	

Severe nausea, pain, HIV infection	

Idiopathic	

*Nohypothalamic* (*ectopic*)* production of ADH *	

Carcinoma: small cell carcinoma of lung, pancreas, thymus, prostate, uterus …	

Lymphosarcoma, mesothelioma	

Hodgkin's disease, leukemia	

Potentiation of ADH effect:	
(i) chlorpropamide	
(ii) carbamazepine	
(iii) psychosis	
(iv) prostaglandin-synthesis inhibitors (salicylates, NSAID)	

Exogenous administration of ADH	
